# Retinal editome profiling in mice reveals RNA editing in key developmental genes associated with retinitis pigmentosa

**DOI:** 10.7717/peerj.21019

**Published:** 2026-05-15

**Authors:** Chun-Yan Ren, Yi-Chen Zhang, JinPing Yao, Tao Guo, Lei Chang, Jian-Huan Chen, Yanshan Liu

**Affiliations:** 1 Laboratory of Genomic and Precision Medicine, Wuxi School of Medicine, Jiangnan University, Wuxi, Jiangsu, China; 2 Department of Pediatric Laboratory, Affiliated Children’s Hospital of Jiangnan University (Wuxi Children’s Hospital), Wuxi, Jiangsu, China; 3 Department of General Surgery, Affiliated Hospital of Jiangnan University, Wuxi, China; 4 MOE Medical Basic Research Innovation Center for Gut Microbiota and Chronic Diseases, Wuxi School of Medicine, Jiangnan University, Wuxi, Jiangsu, China

**Keywords:** RNA editing, Retinitis pigmentosa, Key genes

## Abstract

**Background:**

Adenosine-to-inosine (A-to-I) RNA editing has been found to function in various neurological disorders; however, the role of A-to-I RNA editing in retinitis pigmentosa (RP) remains unclear.

**Methods:**

RNA editing profiles of mouse retinas at different developmental stages, and three RP mouse models that were sampled at the peak of photoreceptor cell death for each model were analyzed to identify significant RNA editing events and genes involved in development and RP pathogenesis. Data from two addtional RP models were used for validation. Key editing sites were validated by Sanger sequencing and dual-luciferase reporter assays.

**Results:**

Global A-to-I editing levels increased during normal retinal development, correlating with *Adar/Adarb1* expression. In RP models, significant alterations in editing landscapes were observed, including dysregulated editing of 55 IRD-related genes. Functional enrichment and protein-protein interaction (PPI) analyses highlighted 10 hub genes, including * Rgs9bp*, which showed extensive editing and elevated expression. Editing at specific sites in * Rgs9bp* enhanced reporter gene expression, implying a functional impact. Notably, *Rgs9bp*, traditionally linked to cone-specific bradyopsia, exhibited hyper-editing in rod-dominant RP models, suggesting a broader role in retinal degeneration.

**Conclusions:**

Our study reveals that A-to-I RNA editing is dynamically regulated during retinal development and profoundly altered in RP, implicating RNA editing as a novel layer of gene regulation in inherited retinal diseases.

## Introduction

RNA editing is a widespread post-transcriptional process that occurs both in the nucleus and mitochondria, wherein the nucleotide sequences of RNA are altered to modulate gene expression and function (Brennicke, Marchfelder & Binder, 1999). The adenosine-to-inosine (A-to-I) conversion, catalyzed by the enzymes of the adenosine deaminase acting on RNA (ADAR) family, is one of the most prevalent RNA modifications found in the mammalian transcriptome (Gu et al., 2012; Rodriguez Morales et al., 2023). Dysregulation of A-to-I RNA editing has been increasingly implicated in the pathogenesis of a range of disorders, particularly rare hereditary and neurodegenerative neurological diseases, as well as immune-related conditions (Rice et al., 2012; Tassinari et al., 2023; Eisenberg & Levanon, 2018).

Retinitis pigmentosa (RP, MIM 268000) is one of the major forms of inherited retinal disease (IRD) (Botto et al., 2022; Zhang, 2016). It is characterized by the gradual degeneration of rod and cone photoreceptors, along with atrophy of the retinal pigment epithelium (RPE), ultimately resulting in irreversible loss of vision (Verbakel et al., 2018). RP exhibits marked genetic heterogeneity, with more than 70 causative genes for RP have been reported. Nevertheless, the mechanisms underlying RP pathogenesis remain incompletely understood. There is no curable method for RP so far, though some exploratory treatments have achieved phased progress. For example, targeted delivery of defective genes through intravitreal injection of recombinant adeno-associated virus (AAV) vectors, or allogeneic iPSC-derived retinal organoid transplantation have demonstrated significant improvement of visual function lasting up to 24 months in clinical trials (Hirami et al., 2023; Yang et al., 2025). However, the long-term efficacy and side effects of these methods remain unclear. Moreover, the high genetic heterogeneity of RP severely limits the development of precision treatment strategies. Therefore, a thorough elucidation of the molecular mechanisms underlying RP, genetically and epigenetically, holds significant scientific value.

Genetically engineered RP mouse models, such as *Pde6b*^rd1^ (rd1), *Pde6b*^rd10^ (rd10) mice, and *Prph*^rd2^ (rd2) mice, provide important tools for studying the underlying pathophysiological basis of RP. The rd1 mice exhibit early-onset severe retinal degeneration due to a homozygous nonsense mutation in exon 7 of the Phosphodiesterase 6B (*Pde6b*) gene (Chang et al., 2002). The rd2 mice, also known as retinal degeneration slow (RDS), harbor a homozygous mutation in the Peripherin 2 (*Prph2*) gene, leading to the loss of photoreceptor outer segments (Goldberg, 2006). The rd10 mice model carries a homozygous missense mutation in the *Pde6b* gene, which affects the catalytic subunit of protein and results in significant reduction of its enzyme activity (Wei et al., 2021). Importantly, recent studies suggest that epigenetic modifications, in addition to canonical genetic variations, contribute to retinal diseases (Popova et al., 2021). Through a comprehensive analysis of RNA editing in the human retina using post-mortem samples from over 500 donors, including those with age-related macular degeneration (AMD), Ansell et al. (2023) identified thousands of edited sites, characterized retinal editing quantitative trait loci (edQTLs), and showed that RNA editing variation colocalized with genetic risk loci for several retinal diseases. However, the potential role of A-to-I RNA editing in the development or progression of RP remains largely unexplored.

In the present study, we comprehensively profiled the RNA editome in the developing mouse retina and three well-established RP mouse models. Our analysis revealed dynamic A-to-I RNA editing events in genes related to retinal development, hinting that dysregulation of RNA editing may contribute to RP pathogenesis and offering novel insights into the molecular mechanisms underlying this disorder.

## Materials and Methods

### RNA-seq dataset retrieval

The RNA-seq raw data utilized in this study were obtained from the European Nucleotide Archive (ENA, URL: https://www.ebi.ac.uk/ena) hosted by the European Molecular Biology Laboratory. The discovery dataset PRJNA396074 (Brooks et al., 2019) includes transcriptomic analyses of mouse retinas at four embryonic stages (E11, E12, E14, E16) and eight postnatal stages (P0, P2, P4, P6, P10, P14, P21, P28), each stage contains two biological replicates. Dataset PRJNA741503 contains retinal RNA-seq from three RP mouse models along with wild-type C3H mice. Retinas were sampled at the peak of photoreceptor cell death for each model, rd1 mice at postnatal day 13 (P13), rd10 mice at P23, and rd2 mice at P29, respectively (Wei et al., 2021). Each model contains three biological replicates. The validation RNA-Seq data in PRJNA243383 contains retina samples from rd10 mice, wild-type mice, and GFP-expressing mice, with three biological replicates (Uren et al., 2014). The RNA-seq dataset PRJNA892591 includes experimental, control, and wild-type groups. The experimental group consists of Rds/rd2 retinas treated with lentivirus overexpressing CNTF, the control group consists of rd2 retinas treated with lentivirus expressing GFP, and the wild-type group is untreated (Do Rhee et al., 2022).

### Alignment of RNA sequencing data

RNA sequencing read processing and RNA editing events identification were performed as previously described in our lab (Zhang et al., 2022). Briefly, the sequencing data were subjected to initial quality control using FastQC upon retrieval. Adapter sequences and low-quality reads were eliminated using fastp (Version 0.23.4) after retrieval (Chen et al., 2018). RNA STAR (Version 2.7.0e) was then used to align the sequencing reads to the Mus musculus reference genome (UCSC mm10) (Dobin & Gingeras, 2015), generating binary alignment map (BAM) files. SAMtools (Version 1.9) was used to process the BAM files, eliminating optical duplicates and retaining only the reads aligned to the Mus musculus reference genome (Li et al., 2009). Lastly, GATK (Version 4.1.3) was used to recalibrate the quality scores of the BAM files, following the best practices outlined in the official documentation (Der Auwera et al., 2013).

### Identification of high-confidence A-to-I RNA editing events

VarScan (Version 2.4.3) was used to detect Single Nucleotide Variants (SNVs) from the BAM files (Koboldt et al., 2012). The variant calling criteria included a minimum base quality of 25, a total sequencing depth of ≥ 10, an alternative allele depth of ≥ 2, and an Alternative Allele Frequency (AAF) of ≥ 1%. The default filtering parameters of VarScan were used to reduce the occurrence of false-positive SNVs. SNVs were annotated using the Ensembl Variant Effect Predictor (VEP) tool (McLaren et al., 2016). Further filtering was applied, except for SNVs that were annotated as RNA editing sites in the REDIportal V2.0 database. SNVs were excluded if they satisfied any of the following conditions: (1) Found within homopolymer runs (≥ 5 nucleotides), simple repeats, or the mitochondrial genome. (2) Located ≤ 6 nucleotides from splice junctions, ≤ 1 nucleotide from insertions or deletions, or within 4% of the sequencing read ends. (3) Previously listed in the dbSNP database (Build 142). (4) Showing AAF = 100% or 40% ≤ AAF ≤ 60% in over 90% of the samples (Zhang et al., 2022; Pan et al., 2023). High-confidence A-to-I RNA editing events (detected as A-to-G transitions in RNA-Seq reads) were retained if editing levels were ≥1% in at least two samples (Mansi et al., 2021).

### Gene expression quantification analysis

Gene expression levels were quantified using FeatureCounts (Version 2.0.1) to calculate pseudo-counts from BAM files. Subsequently, the Transcripts Per Kilobase Million (TPM) values for each gene were obtained using EdgeR (Version 3.7) (McCarthy, Chen & Smyth, 2012).

### Gene function enrichment analysis

We performed Gene Ontology (GO) and Kyoto Encyclopedia of Genes and Genomes (KEGG) pathway enrichment analysis of edited genes using Enrichr (Kuleshov et al., 2016) and metascape (Zhou et al., 2019) to better understand the functional relevance of the associated genes. Items with *p*-values < 0.05 were considered significant.

### Statistical analysis

We used the Generalized Linear Model (GLM) and Likelihood Ratio Test (LRT) to compare RNA editing levels across groups to identify differential RNA editing (Pan et al., 2023). Gene expression levels were compared using the Student’s t-test. Spearman’s correlation method was then used to calculate the correlation coefficient (r) and *p*-values, followed by the analysis of the correlation between RNA editing and gene expression (Kong et al., 2023). Principal Component Analysis (PCA) of A-to-I RNA editing events was conducted using R (Version 3.6.3; R Core Team, 2020) (17) to assess the contribution of RNA editing to the differences between various retinal degeneration (RD) mouse models and controls.

### Confirmation of RNA editing sites in mouse retina RNA with Sanger sequencing

For the confirmation of RNA editing sites, retina samples from three healthy male C57BL/6 mice (8–12 weeks) were collected. The experiment was conducted in accordance with the ethical guidelines approved by the Animal Welfare Committee of Wuxi School of Medicine, Jiangnan University (JN. No20231215m0500630). The mice were obtained from Cyagen, China. Prior to euthanasia, the animals were housed in a temperature-controlled environment (24 ± 1 °C) with a 12-hour light/dark cycle, and they had *ad libitum* access to food and water. Mice were housed in cages measuring 290 mm  ×  178 mm  ×  160 mm (Length × Width × Height), with 4–5 mice per cage to ensure adequate space for activity and social needs.

Mice scheduled for sampling were anesthetized using isoflurane (0.5 ml/dm^3^), followed by euthanasia *via* cervical dislocation. Death was confirmed by observing indicators such as respiration, heartbeat, pupil response, and neural reflexes. The deceased mice were perfused with pre-cooled (4 °C) physiological saline to flush the systemic circulation. The eyeballs were then enucleated. The cornea was cut along the corneoscleral limbus of the eyeball. Using forceps, the lens was gently inserted and slowly pulled outward, allowing the milky-white retina to detach along with the lens. By rotating the eyeball, the entire lens was slowly extracted, and the retina was completely detached as well. The lens and retina were then separated. Any residual black iris tissue at the edges of the retina was carefully removed. Finally, the retina was dissolved.

For each mouse, retinas from the left and right eyes were used separately for genomic DNA and total RNA extraction, respectively. DNA and RNA extraction was performed with TIANamp Genomic DNA Kit (Cat. No. DP304) and RNA Simple Total RNA Extraction Kit (Cat. No. DP419) from TIANGEN (TIANGEN, China), respectively. The concentration and purity of the extracted DNA and RNA samples were measured using a spectrophotometer to ensure their quality met the requirements for subsequent experiments. Reverse transcription was then performed using the HiScript^®^ III RT SuperMix for qPCR (+ gDNA wiper) kit from Nanjing Vazyme Biotech Co., Ltd. to synthesize complementary DNA (cDNA).

Primers targeting four differential RNA editing sites (*Rgs9bp*: chr7:35581718, *Rgs9bp*: chr7:35581747, *Padi2*: chr4:140951612 and *Calm1*: chr12:100201186) were designed to test the editing at both DNA and RNA levels (Table 1). Polymerase chain reaction (PCR) reactions were conducted using the 2 ×Taq MasterMix kit (Cat. No. CW0682L) from CoWin Biotech Co., Ltd. to enhance amplification efficiency and specificity. The amplified products were verified by agarose gel electrophoresis and subsequently sent to Shanghai Sangon Biotech Co., Ltd. for Sanger sequencing. Upon receiving the sequencing data, SnapGene software (version 6.0.2) was used for sequence analysis and alignment to confirm the accuracy of the target sequence. At least three retinas from different mice were sequenced for confirmation.

**Table 1:** Primer pairs used for confirmation of RNA editing sites.

Primer_name	Sequences
*Rgs9bp*-F	5′-AGTTGGAGCGCGAAGTCCTC-3′
*Rgs9bp*-R	5′-GAGGGACCTGGGGTCACTGT-3′
*Padi2*-F	5′-TCCACGGGCTTCTGCACAT-3′
*Padi2*-R	5′-AGATGTGCGCTTTGTCTGCA-3′
*Calm1*-F	5′-TGGTTTACCTCAGGATGATTGATG-3′
*Calm1*-R	5′-GAGTCIGTCTGGTTTCAGGAGTTG-3′

### Validation of functional outcome of RNA editing sites with Dual Luciferase analysis

Luciferase analysis was performed to assess the functional impact of RNA editing sites from *Rgs9bp* (*Rgs9bp*: chr7:35581718, *Rgs9bp*: chr7:35581747) and *Padi2* (*Padi2*: chr4:140951612) on RNA expression as previously described (Kong et al., 2024). Briefly, psiCHECK-2 vector (Promega, Madison, WI, USA) was used as backbone for reporter construct generation. Fragments containing either the wild type (WT) or edited-site were synthesized and cloned into the multiple cloning sites downstream of the Renilla luciferase. The resulting constructs were transfected into the Human embryonic kidney (HEK293T) using Lipofectamine 3000 (Thermo Fisher Scientific, Waltham, MA, USA), following the manufacturer’s protocol. 48 h after transfection, cells were harvested and assayed for both firefly and Renilla luciferase activities with Dual-Luciferase Reporter System kit (Cat No. DL101-01) from Vazyme according to manufacturer’s instructions (Vazyme). Each experimental condition was measured six times.

## Results

### Increased A-to-I editing activities in the mouse retina during development

**Figure 1:**
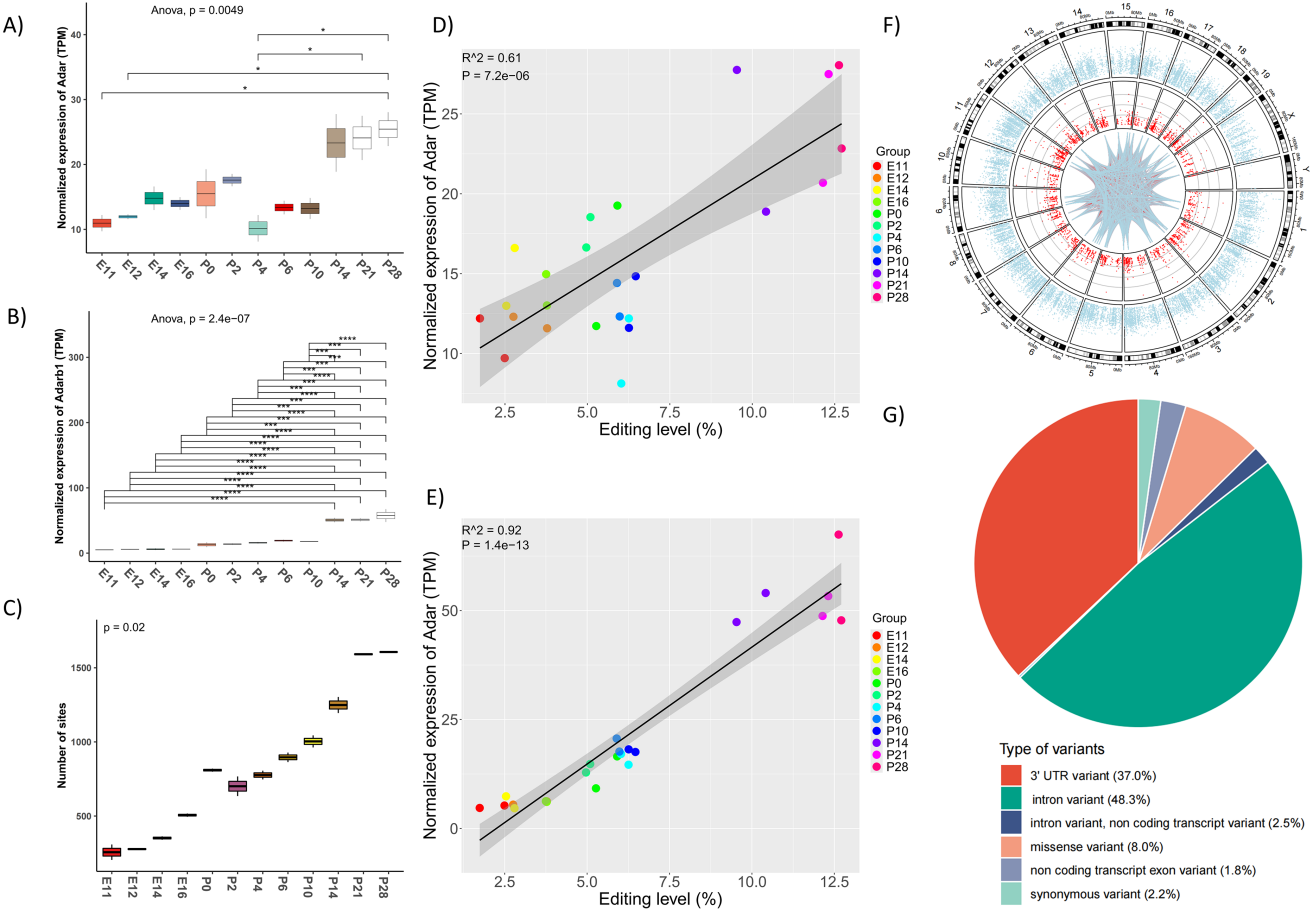
Increased A-to-I editing activities in the mouse retina during development. (A) and (B) The expression of *Adar* and *Adarb1* upon different time points. * *P* < 0.05, **** *P* < 0.001. (C) The number of RNA editing sites detected in different time points. (D) and (E) Correlation analysis between the expression levels of *Adar* (D) and * Adarb1* (E) with the overall RNA editing levels, data and graphs were generated with linear regression and correlation analysis by R package ggplot2. (F) Spearman correlation plot that was calculated with the RNA editing levels of all genes from different time points. The analysis was performed and generated with R package ggplot2 and reshape2. (G) The circos plot showed the distribution of RNA editing sites across mouse chromosomes. And the lines denote interaction between the RNA editing events, with the blue-to-red gradient indicated the correlation co-efficient r. The interaction among the top 100 frequently observed editing events is shown. (H) Functional annotation of RNA editing sites.

To gain a comprehensive understanding of RNA editing dynamics in the developing mouse retina, we profiled the A-to-I RNA editome during multiple retina developmental time points (14). The expression of the two major A-to-I RNA editing enzymes, *Adar* and *Adarb1*, progressively increased throughout retinal development (Figs. 1A, 1B). Consistent with the upregulation, the total number of edited sites, genes, and overall editing levels all exhibited significant increases over time (Fig. 1C, Figs. S1A, S1B). Further analysis revealed a positive correlation between editing levels and the expression levels of *Adar* and *Adarb1* (Figs. 1D, 1E).

In total, we identified 2,820 high-confidence A-to-I RNA editing sites from 1,175 genes (Fig. 1G). Further alignment and functional annotations of these editing sites showed most of the sites were in intron (48.3%) and 3’ untranslated regions (3’UTR) (37%) of edited genes (Fig. 1H).

### RNA editing could regulate key gene expression during development

**Figure 2:**
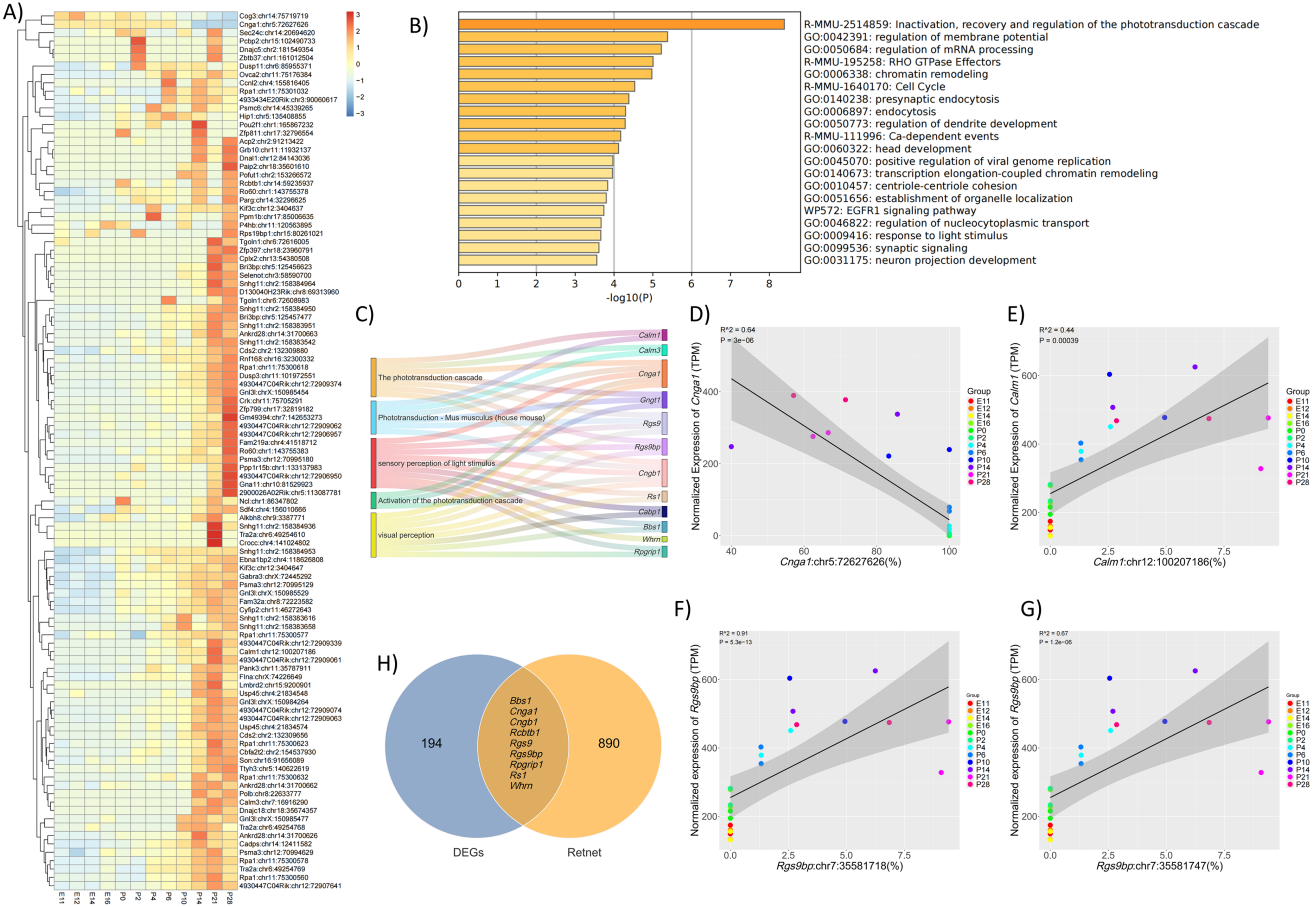
RNA editing could regulate key gene expression during development. (A) Heatmap showed the top differentially edited sites (DESs) in normal mouse retina. (B) Bar plot showed the functional enrichment analysis with differentially edited genes (DEGs). (C) Sankey plot indicated the DEGs that involved in phototransduction related pathways. (D, E, F) and (G) showed the correlations between the expression levels of Cnga1, Calm1 and Rgs9bp with the editing levels of Cnga1:chr5:72627626, Calm1:chr12:100207186, Rgs9bp:chr7:35581747 and Rgs9bp:chr7:35581718, respectively. (H) Venn diagram showed the overlaps between DEGs and genes related to IRDs.

We utilized the GLM to identify the differentially editing sites (DESs) and differentially edited genes (DEGs) across the sampled developmental time points. In total, 1,436 DESs corresponding to 670 DEGs were identified. Consistent with the global editing trends, the majority of DESs exhibited a progressive increase in editing levels, with the peak activity observed at the last three time points (Fig. 2A, Table S1). Moreover, Spearman correlation analysis identified 380 DESs, spanning 203 genes that showed significant correlations between their editing levels and expression levels of their host genes, suggesting a potential regulatory interplay between A-to-I RNA editing and gene expression. Functional enrichment analysis of these DEGs highlighted involvement in retinal-specific pathways, including the phototransduction cascade, RHO GTPase effectors, response to light stimulus, and so on (Fig. 2B).

We then focused on the DEGs implicated in retinal-related pathways. Several DEGs were identified in the aforementioned functional categories, such as *Calm1*, *Cnga1*, *Rgs9bp*, and so on (Fig. 2C). Notably, RNA editing levels at specific sites within these genes, such as *Calm1* at Chr12:100207186, *Cnga1* at Chr5:70627626, *Rgs9bp* at Chr7:35581747 and Chr7:35581718, and others showed significant correlations with their respective expression levels (Figs. 2D–2G). Moreover, we cross-referenced our DEG list with genes in RetNet (https://retnet.org/), a database recording genes and loci associated with IRDs, which revealed nine overlapped genes. Diseases related to these genes contained Bardet-Biedl syndrome (*BBS1*), retinitis pigmentosa (*BBS1*, *CNGA1*, *CNGB1*), retinal dystrophy (RCNTB1), recessive delayed cone adaptation (*RGS9BP*), Leber congenital amaurosis (*RPGRIP1*) and so on, suggesting that RNA editing may contribute to the post-transcriptional regulation of these genes, potentially influencing their expression and function in retinal pathophysiology (Fig. 2H).

### Dynamic regulation of A-to-I RNA Editing in RP mouse models correlated with *Adar*/*Adarb1* expression

**Figure 3:**
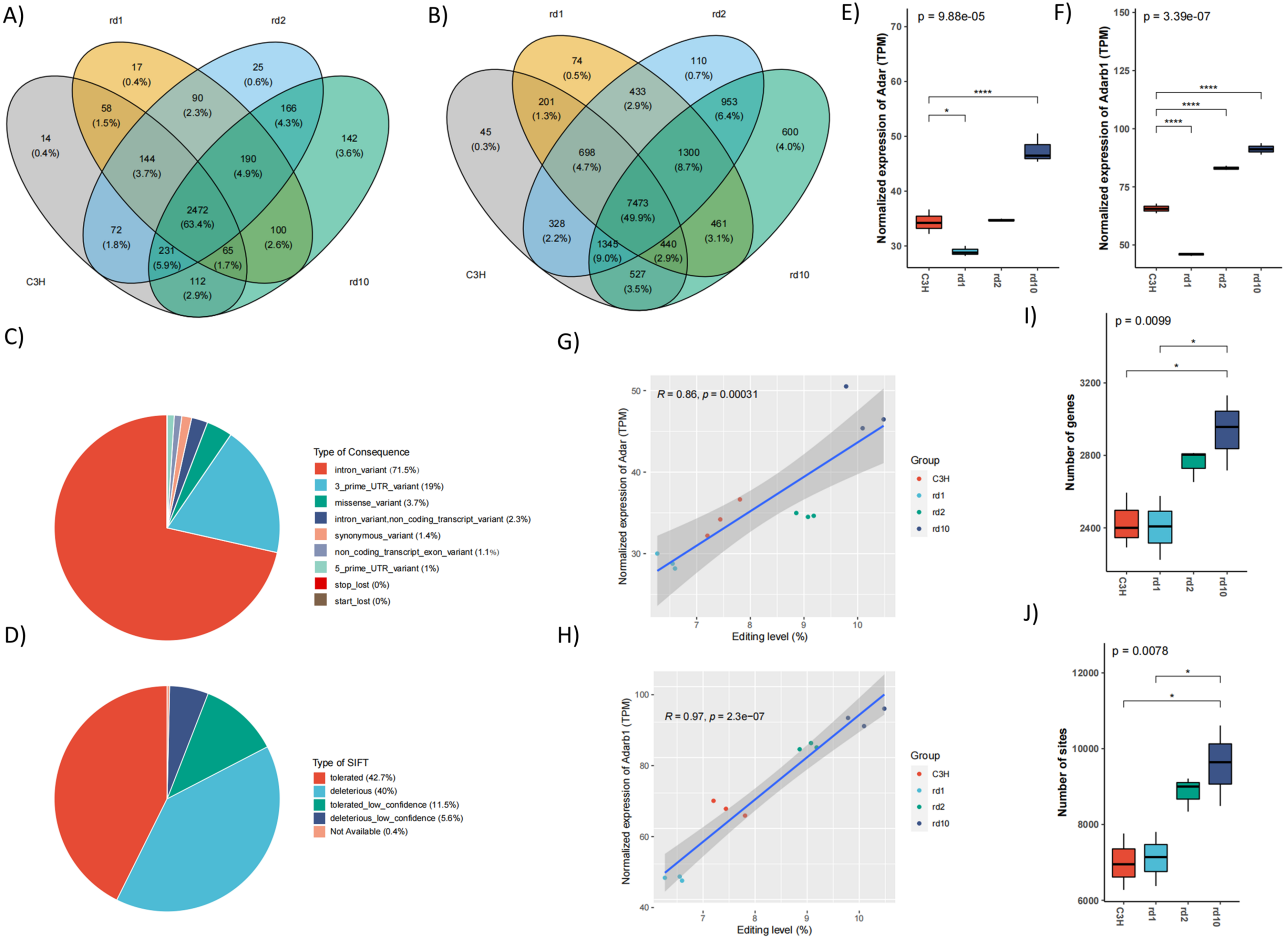
RNA editing was altered in different RP mouse models. (A) and (B) Venn diagram indicated the comparison among normal mouse retina (CH3) and three different mouse models (rd1, rd2 and rd10) with regard to editing sites and genes. (C) Functional annotation of RNA editing sites identified within our analysis. (D) Prediction of functional outcomes of RNA editing sites that resulted in missense mutations. (E–F) The expression of *Adar* and *Adarb1* in different mouse models. * *P* < 0.05, **** *P* < 0.001. (G–H) Correlation analysis between the expression levels of *Adar* (D) and *Adarb1* (E) with the overall RNA editing levels. (I–J) The number of RNA editing sites and genes detected in different mouse models.

To investigate the role of RNA editing in RPs, we performed a comprehensive editome analysis using dataset PRJNA741503, which contains RNA-seq data from three RP mouse models, rd1, rd2, and rd10. A total of 14,988 high-confidence A-to-I RNA editing sites in 3,898 genes in the retinas of control and three RP mouse models were identified. In control C3H, 14 genes exhibited unique editing, whereas rd1, rd2, and rd10 models showed unique editing in 17, 25, and 142 genes, respectively (Fig. 3A). Similarly, unique editing sites were observed in each group, with 45 sites exclusive to C3H, while rd1, rd2, and rd10 had 74, 110, and 600 unique sites, respectively (Fig. 3B). Genomic annotation of all detected editing sites revealed that the majority (71.5%) resided in intronic regions, with an additional 19.0% located in 3’UTRs (Fig. 3C), consistent with the canonical distribution of A-to-I editing events in mammals.

Compared to the control group, we observed a reduced expression of *Adar* and *Adarb1* in the rd1 retina, whereas their expression was elevated in both rd2 and rd10 (Figs. 3E, 3F). Consistent with these expression patterns, a positive correlation was observed between the expression of these two enzymes and overall RNA editing levels (Figs. 3G, 3H). Furthermore, the number of edited genes and sites was higher in rd2 and rd10 compared to both control and rd1 samples (Figs. 3I, 3J). Together, these results suggested that RNA editing was dynamically regulated in RP mouse models, and these alterations closely paralleled the expression levels of key editing enzymes, particularly *Adar* and *Adarb1*.

### Identification of 10 hub DEGs potentially involved in RP pathogenesis

**Figure 4:**
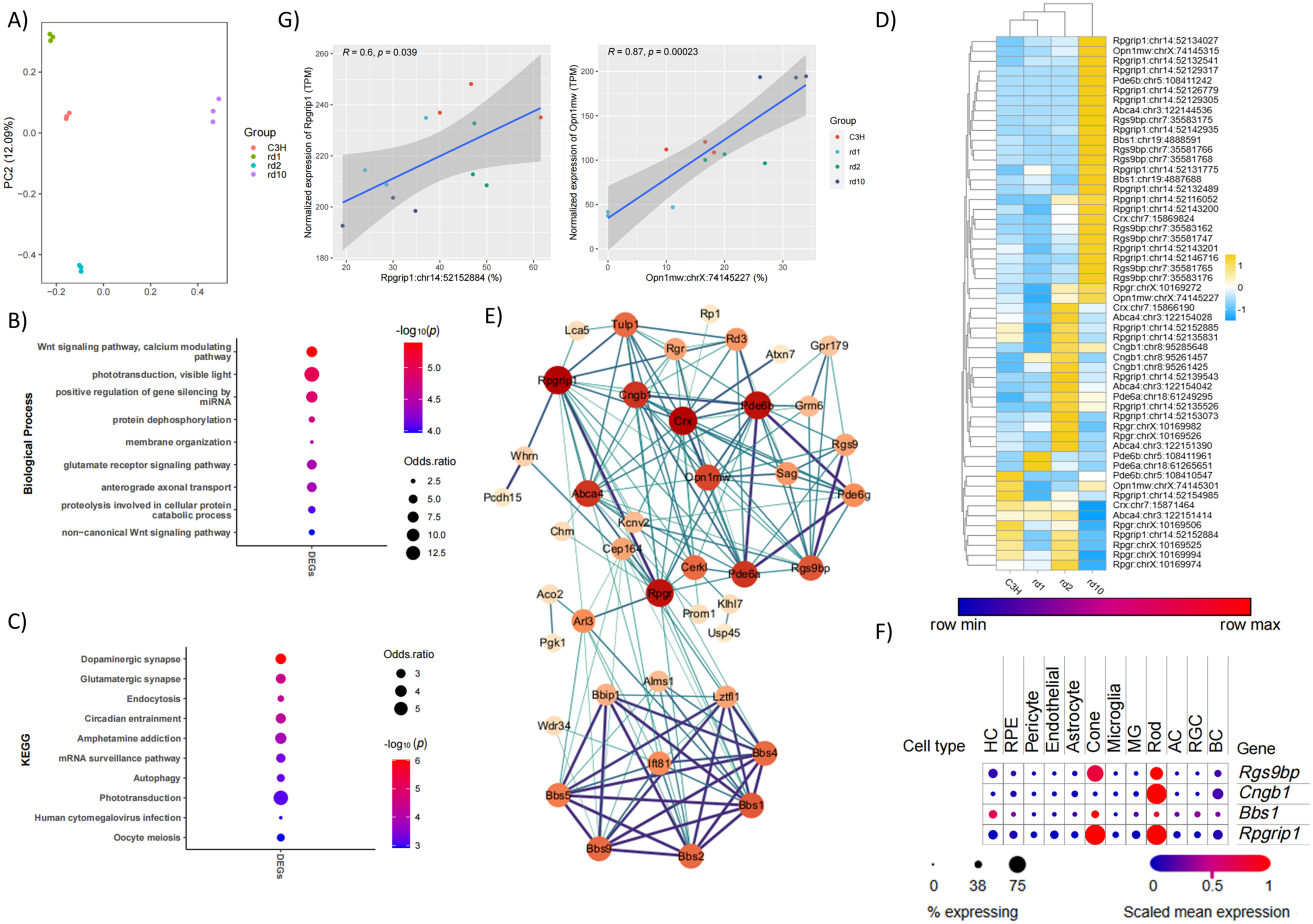
Identification of 10 hub DEGs potentially involved in RP pathogenesis. (A) Principal component analysis (PCA) showed the separation of different mouse models. (B) and (C) GO and KEGG analysis with DEGs identified in our analysis. (D) Visualization of protein-protein interaction network of overlapped genes. The node size represents the number of direct neighbors, and the thickness and color of the edges represent the enrichment score. (E) Heatmap indicated the DESs found in hub genes. (F) Dot plot showed the expression of Rgs9bp, Cngb1, Bbs1 and Rpgrip1 in different cell types of mouse retina. The size of dots represent the percentage of expressed cells in certain cell type, while the blue to red gradient indicated the mean expression of genes in certain cell types.

To uncover distinctive RNA editing patterns associated with RP, we performed comparative analyses between control and RP mouse models. Principal component analysis (PCA) of these differential RNA editing sites showed that PC1 and PC2 explained 12.09% and 57.82% of the total variance, respectively (Fig. 4A).

A total of 3,788 DESs from 1,725 DEGs were identified (Table S1). Functional enrichment analysis of overall DEGs showed overrepresentation of RP-related functional annotations, most notably phototransduction in biological processes (Fig. 4B). Consistent with this finding, KEGG pathway analysis revealed enrichment in RP-related pathways, including phototransduction, autophagy, endocytosis, and so on (Fig. 4C).

A comparison of DEGs and genes from Retnet showed an overlap of 55 genes (Fig. 4D). To further explore their functional interactions, a protein-protein interaction (PPI) network was constructed by utilizing the STRING database (https://cn.string-db.org/), which demonstrated strong connectivity among these genes. From the PPI network, we identified 10 hub genes with at least 12 interactions, including *Crx* (17), *Rpgrip1* (17), *Pde6b* (16), *Rpgr* (16), *Abca4* (14), *Cngb1* (14), *Pde6a* (14), *Opn1mw* (13), *Bbs1* (12) and *Rgs9bp* (12). These genes were both differentially edited and strongly implicated in RP pathogenesis. Across these hub genes, 54 DESs were identified in total (Fig. 4E), and several of these sites exhibited a significant correlation between RNA editing levels and gene expression, indicating their potential functional relevance.

Among these hub genes, *Bbs1*, *Cngb1*, *Rgs9bp,* and *Rpgrip1* were found both in RP models and in the normal developmental retina. To visualize the expression of the four genes across various cell types in the mouse retina, we utilized data from the Single Cell Portal (https://singlecell.broadinstitute.org/single_cell). Only a small percentage of rod and cone photoreceptor cells expressed *Bbs1.* In contrast, *Cngb1* had high expression in rod cells but not in cone cells. The other two genes, *Rgs9bp* and *Rpgrip1*, showed relatively high expression in both rod and cone cells (Fig. 4F).

*RPGRIP1* is well-known as a pathogenic gene for RP. Notably, *Rgs9bp*, whose variations were found to be responsible for delayed cone adaptation to sudden light changes or bradyopsia (Strauss et al., 2015). However, our findings suggest that *Rgs9bp* may also play a role in RP, a disease characterized by initial rod photoreceptor degeneration.

### Validation across datasets confirmed consistent RNA editing in RP models

**Figure 5:**
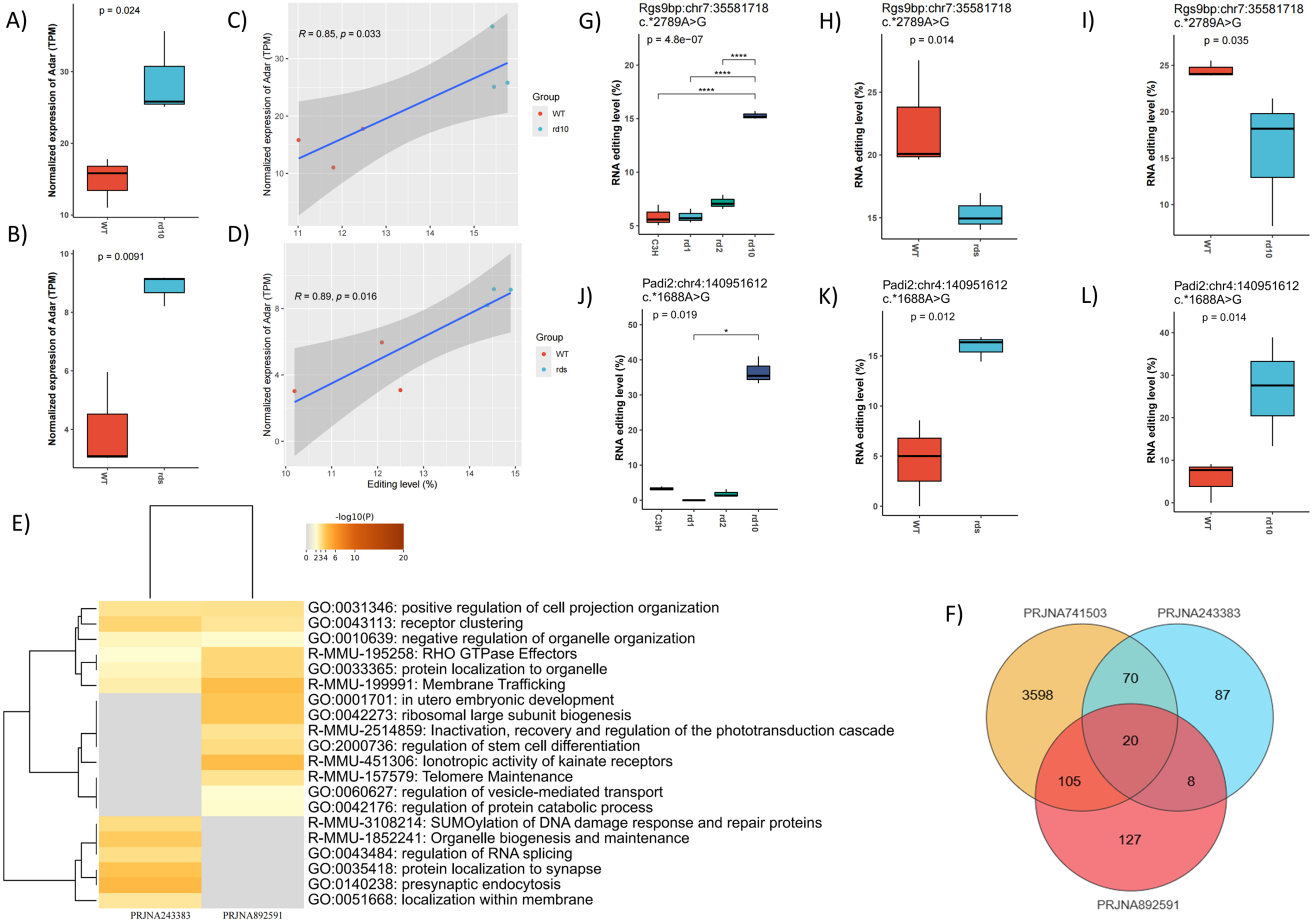
Validation across datasets confirms consistent RNA editing in RP models. (A) and (B) The expression of *Adar* in validation datasets, PRJNA243383 (WT *vs* rd10) and PRJNA892591 (WT *vs* rds), respectively. (C) and (D) Correlation analysis between the expression levels of *Adar* with the overall RNA editing levels of PRJNA243383 and PRJNA892591. (E) Functional annotation of DEGs found in PRJNA243383 and PRJNA892591. (F) Venn diagram illustrated the overlaps between DESs in different datasets. (G, H) and (I) showed the editing levels of Rgs9bp:chr7: 35583175 in PRJNA741503 (G), PRJNA243383 (H) and PRJNA892591 (I), respectively. J), K) and L) showed RNA editing levels of Padi2:chr4:140951161 in PRJNA741503 (J), PRJNA243383 (K) and PRJNA892591 (L), respectively.

To confirm such findings, we incorporated two validation datasets, PRJNA243383 and PRJNA892591, which contained the rd10 and rd2 mouse models, respectively. Differential expression of the two main enzymes was found (Figs. 5A–5B). Moreover, we found that the overall RNA editing levels showed a significant correlation with the expression of at least one RNA editing enzyme (Figs. 5C–5D). The identified editing sites were 1,082 and 1,795 for PRJNA243383 and PRJNA892591, coming from 504 and 773 genes, respectively. 433 sites from 205 genes were shared among all datasets, and 1,098 sites from 562 were shared by at least two datasets.

185 and 260 DESs were identified from PRJNA243383 and PRJNA892591, respectively. Functional analysis with DEGs suggested enrichment of retina-related pathways, such as RHO GTPase effectors, membrane trafficking and so on (Fig. 5E). Comparison with PRJNA741503 showed 20 common DESs in all three datasets (Fig. 5F). Four genes had more than two DESs, including *Rgs9bp* and *Padi2*, each harboring three editing sites (Figs. 5G–5L). Differential editing of *Rgs9bp* was consistently detected across all tested RP mouse models, with three to eight DESs per model (Table S2), hinting at its potential role as a convergent node in the post-transcriptional response to retinal stress.

### Functional validation of RNA Editing Sites confirmed their role in regulating host gene expression

**Figure 6:**
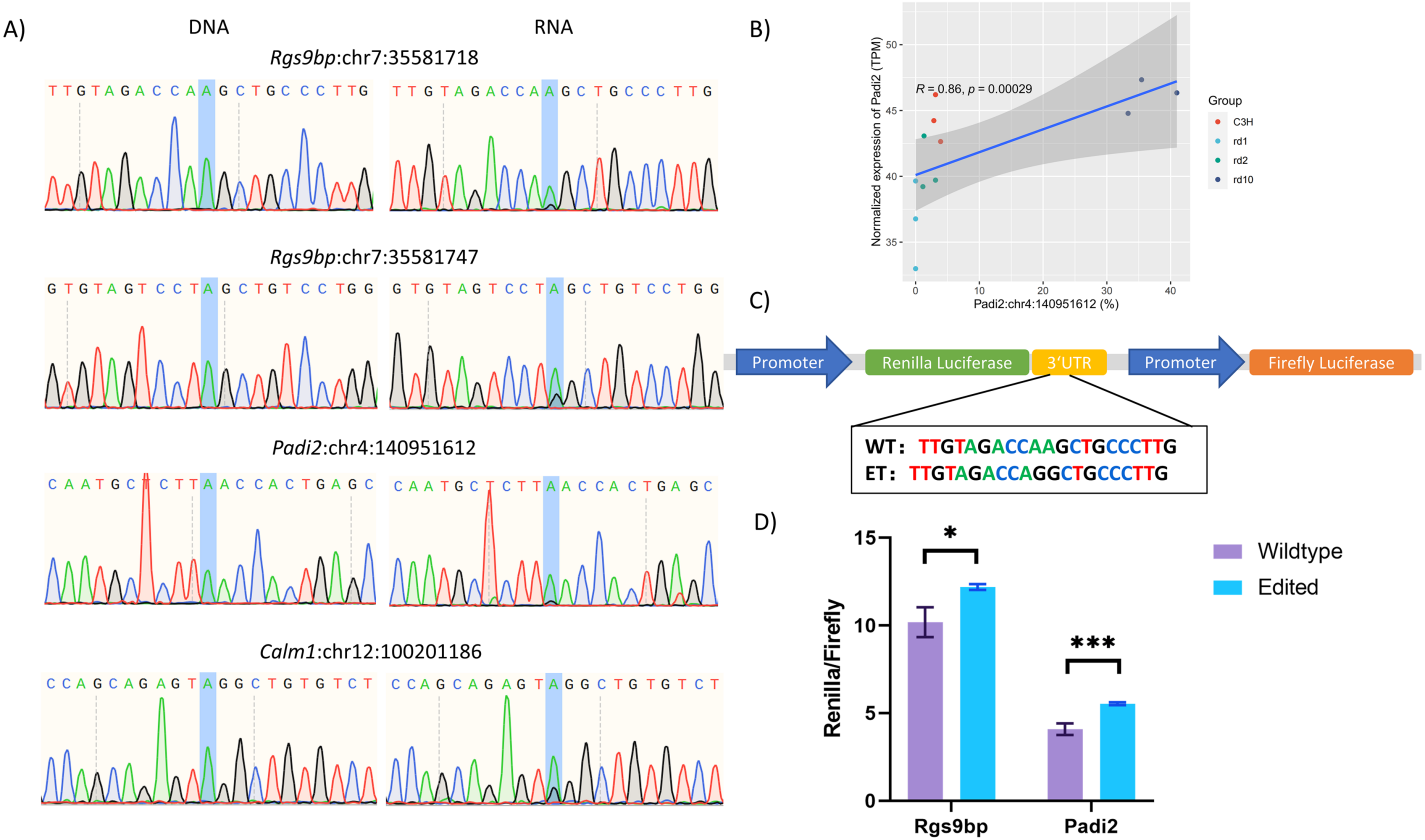
Validation of RNA editing sites and their functional outcome with Dual Luciferase analysis. (A) Confirmation of DESs with Sanger sequencing. Chromatogram showed sequencing results of four selected RNA editing sites in cDNA and corresponding genomic DNA. (B) The correlation between the expression levels of *Padi2* with the editing levels of *Padi2*:chr4:140951612 in control and three RP mouse models. (C) Schematic diagram showed the usage of Dual-luciferase assay to validate the functional outcome of tested RNA editing sites. (D) Dual-luciferase assays of wild-type compared to the edited-type 3′-UTR of *Rgs9bp* and *Padi2*. *P < 0.05, ***P < 0.001.

To validate the existence of these DESs, we performed Sanger sequencing on matched genomic DNA and RNA samples of mouse retina. Four editing sites from three genes were selected for confirmation (Fig. 6A, Fig. S2). No nucleotide alterations were detected in DNA samples, confirming the absence of underlying genetic variants. In contrast, A-to-G conversions, which reflected A-to-I editing at RNA level, were consistently observed in cDNA samples across all tested retinas, albeit at varying editing efficiencies.

We selected three A-to-I RNA editing sites for functional validation based on our bioinformatic predictions that editing at these sites may modulate host gene expression: *Rgs9bp* (chr7:35581718), *Rgs9bp* (chr7:35581747), and *Padi2* (chr4:140951612) (Figs. 2F, 2G, and 6B). For each site, genomic fragments containing either the wild-type (WT) or edited (A-to-G) sequence were cloned into the multiple cloning site of the psiCHECK-2 reporter vector, positioned downstream of the Renilla luciferase gene (Fig. 6C).

Dual-luciferase assays were performed in HEK293T cells, and the Renilla/Firefly luciferase activity ratio was used to assess the impact of RNA editing on post-transcriptional regulation. Consistent with our computational predictions, the edited constructs exhibited significantly higher Renilla luciferase activity compared to their WT counterparts (Fig. 6D), suggesting that A-to-I editing at these sites enhances the expression or stability of the reporter transcript, thereby supporting a functional role for these editing events in regulating host gene expression.

## Discussion

RP is a hereditary retinal disorder marked by the gradual deterioration of vision and considerable changes in the retinal pigment epithelium (RPE). It often begins with night blindness in its early stages and can advance to complete blindness in severe cases, involving degeneration of both photoreceptor cells (rods and cones) and RPE cells (Dias et al., 2018). RP affects roughly one in every 4,000 individuals worldwide. Despite its relatively high prevalence, effective treatment options remain limited (Verbakel et al., 2018). Consequently, unraveling its underlying pathogenic mechanisms is critical for advancing diagnostic and therapeutic strategies.

Previous studies have indicated a correlation between A-to-I RNA editing and various neurodegenerative diseases (Wu et al., 2023; Wu et al., 2021; Hosaka et al., 2019). In this study, we provide a comprehensive analysis of the A-to-I RNA editome in both developing wild-type mouse retina and three established RP mouse models. We observed dynamic alterations in RNA editing during normal retinal development, with an increase in RNA editing levels in parallel with increased expression of *Adar* and *Adarb1*. Furthermore, analysis of DESs and DEGs showed that numerous genes related to retinal functions exhibited differential RNA editing over time, including nine genes earlier reported to be linked to RP. Further analysis comparing retina samples between control and three different RP mouse models revealed significant differences in RNA editing enzyme expression, the number of edited genes and sites, as well as overall RNA editing levels. These results imply that RNA editing might play a role in retinal development and the pathogenesis of retinal diseases, suggesting its potential as a novel regulatory layer in RP and other inherited retinal diseases.

Notably, we observed distinct RNA editing profiles between rd1 and rd10 mice, two RP models harboring different mutations in the same gene, *Pde6b*. Despite their shared genetic locus, rd10 exhibited markedly higher global editing levels and greater upregulation of *Adar*/*Adarb1* compared to rd1. We speculate that this discrepancy may reflect differences in disease progress and cellular stress responses. The early-onset, aggressive degeneration in rd1 driven by a nonsense mutation may leave little room for adaptive regulatory mechanisms, whereas the delayed onset in rd10 caused by a missense mutation could allow time for stress-induced ADAR activation and compensatory RNA editing. This finding underscores that even allelic variants in the same gene can elicit divergent post-transcriptional landscapes, highlighting the importance of considering mutation-specific effects in both disease modeling and therapeutic development.

In the present study, strong positive correlations between the expression of *Adar* and *Adarb1* and key features of the RNA editome, including the number of edited genes, editing sites, and overall RNA editing levels were observed during retinal development and multiple RP mouse models (Figs. 1D, 1E, 3G, 3H, 5C and 5D). Previous studies have shown elevated *ADARB1* expression in retinal organoids carrying *USH2A* mutant, which may be linked to abnormal processes such as RPE differentiation, polarization, and apoptosis (Guo et al., 2019). The findings suggest that dysregulation of *Adar* and *Adarb1* expression may perturb RNA editing-related signaling pathways, thereby contributing to the molecular and cellular abnormalities underlying retinitis pigmentosa.

By comparing DEGs from different mouse models with IRD-related genes, we identified 55 genes that were differentially edited in mouse models and might contribute to the pathogenesis of IRDs. Ten hub genes were identified through PPI analysis, including *Rgs9bp*. *RGS9* encodes a GTPase-activating protein that plays a key role in regulating retinal phototransduction (He, Cowan & Wensel, 1998). In the retina, RGS9 functions alongside its anchoring protein R9AP, encoded by the *RGS9BP* gene, to facilitate G protein inactivation, thereby terminating light signals and helping restore the cell’s resting state (Nishiguchi et al., 2004). This complex is essential for both rod and cone function, particularly in light adaptation. Loss of function variations in *RGS9* and *RGS9BP* are closely associated with RGS9/R9AP-related retinal degeneration (Michaelides et al., 2010). Clinically, this condition is marked by slow dark and light adaptation, often accompanied by vision loss and abnormal flicker electroretinography responses (Georgiou et al., 2024). These abnormalities typically present in early childhood and share clinical features with other retinal diseases, such as achromatopsia (ACHM) (Strauss et al., 2015). Notably, bradyopsia primarily affects cones, with rods typically spared, distinguishing it from classic RP.

Intriguingly, our study reveals *Rgs9bp* undergoes extensive A-to-I RNA editing in RP mouse models, with elevated expression levels strongly correlated with editing activity during retinal development. The existence and functional outcomes of such sites were further confirmed *via* Sanger sequencing and luciferase assay. Moreover, hyper-editing of *Rgs9bp* was observed across multiple RP models, a disease characterized by initial rod degeneration. This suggests a potential secondary or compensatory role for *Rgs9bp* dysregulation in broader retinal pathology beyond cone-specific dysfunction. While *Rgs9bp* is not traditionally considered an RP-associated gene, our findings implicate RNA editing-mediated modulation of *Rgs9bp* as a potential novel layer of post-transcriptional regulation that may influence disease progression in RP.

In addition to *Rgs9bp*, our functional assays identified *Padi2* as another editing-regulated gene with potential relevance to retinal pathology. We observed A-to-I editing at a specific site within the 3′UTR of *Padi2* (chr4:140951612), and luciferase reporter assays demonstrated that the edited allele significantly enhanced transcript expression compared to the wild-type sequence. *Padi2* encodes peptidyl arginine deiminase 2, which plays a role in the onset and progression of several neurodegeneration disorders, including Alzheimer’s disease and multiple sclerosis (Mondal & Thompson, 2019). *Padi2* levels and global protein deimination decrease with normal aging in rat retina and optic nerve, indicating that reduced deimination is an age-related physiological change (Bhattacharya S.K et al., 2008). In contrast, elevated *PADI2* and protein deimination were observed in age-related ocular diseases like age-related macular degeneration (AMD), glaucoma, and pseudoexfoliation syndrome, suggesting this upregulation is disease-specific, not age-related (Arif & Kato, 2009). Thus, *Padi2* may serve as a potential discriminator between normal aging and disease, implying its potential as a pathological biomarker and therapeutic target in retinal disorders.

Despite the compelling insights provided by our study, several limitations should be acknowledged. First, all analyses were conducted with RP mouse models, which may not fully recapitulate the genetic and phenotypic complexity of human RP. Species-specific differences in RNA editing landscapes, gene expression, or retinal architecture could influence the translatability of our findings to patients. Second, our work relies on publicly available bulk RNA-seq datasets, which may harbor technical artifacts such as batch effects, mapping biases, or incomplete removal of PCR duplicates. Although we implemented stringent quality controls and validated key editing sites experimentally, residual noise or coverage gaps, particularly in lowly expressed genes, could affect editing quantification. Third, while our luciferase assays support a functional impact of editing on *Rgs9bp* expression, direct evidence in photoreceptor cells or *in vivo* models remains lacking. Future studies employing cell-type-specific editing perturbation or patient-derived retinal organoids will be essential to confirm causality and assess therapeutic relevance.

Nevertheless, by integrating developmental and disease-associated editomes across multiple RP models, our work establishes A-to-I RNA editing as a dynamic regulatory layer in retinal homeostasis and degeneration.

In summary, our study provides valuable insights into the role of RNA editing in RP and highlights potential biomarkers and therapeutic targets for further investigation. The heterogeneity of editing landscapes across distinct RP models underscores the molecular complexity of this disease and highlights the need for personalized diagnostic and therapeutic approaches. Importantly, our identification of editing-regulated hub genes, including *Rgs9bp*, offers candidates for biomarker development and RNA-targeted interventions in currently incurable inherited retinal disorders.

##  Supplemental Information

10.7717/peerj.21019/supp-1 Supplemental Information 1 The number of editing sites and editing levels in different time points in normal retinal development The number of editing sites A) and editing levels B) in different time points in normal retinal development.

10.7717/peerj.21019/supp-2 Supplemental Information 2 Confirmation of some DESs in mouse retinas with Sanger sequencing Four DESs (Rgs9bp: chr7:35581718, Rgs9bp: chr7:35581747, Padi2: chr4 :140951612 and Calm1: chr12:100201186) were validated with Sanger sequencing in two other different retina samples at DNA and RNA levels, respectively.

10.7717/peerj.21019/supp-3 Supplemental Information 3 DESs detected in the present study

10.7717/peerj.21019/supp-4 Supplemental Information 4 DESs detected in Rgs9bp from different datasets

10.7717/peerj.21019/supp-5 Supplemental Information 5 ARRIVE 2.0 checklist

10.7717/peerj.21019/supp-6 Supplemental Information 6 Code

## Additional Information and Declarations

### Data Availability

The following information was supplied regarding data availability: The code is available in the Supplemental File. The sequences are available at GenBank: PRJNA396074, PRJNA741503, PRJNA243383, PRJNA892591.
