# Supplementary figures and images for: Retinal editome profiling in mice reveals RNA editing in key developmental genes associated with retinitis pigmentosa
Source: PeerJ. 2026 May 15;14:e21019. doi: 10.7717/peerj.21019 (PMC13182725; doi:10.7717/peerj.21019)

A)

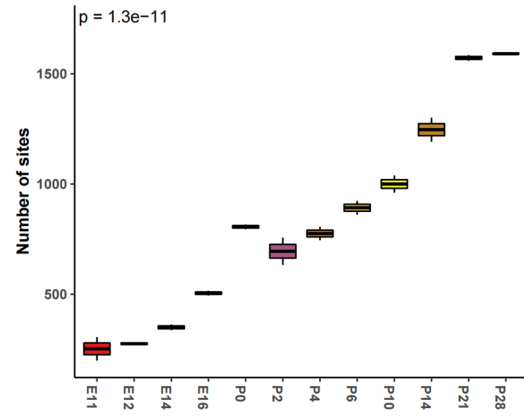

B)

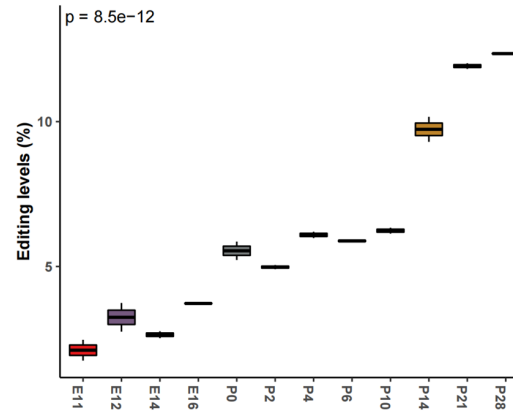

Supplement: Supplemental Information 1 — The number of editing sites A) and editing levels B) in different time points in normal retinal development. [file peerj-14-21019-s001.pdf]

DNA

RNA

*Rgs9bp:chr7:35581718*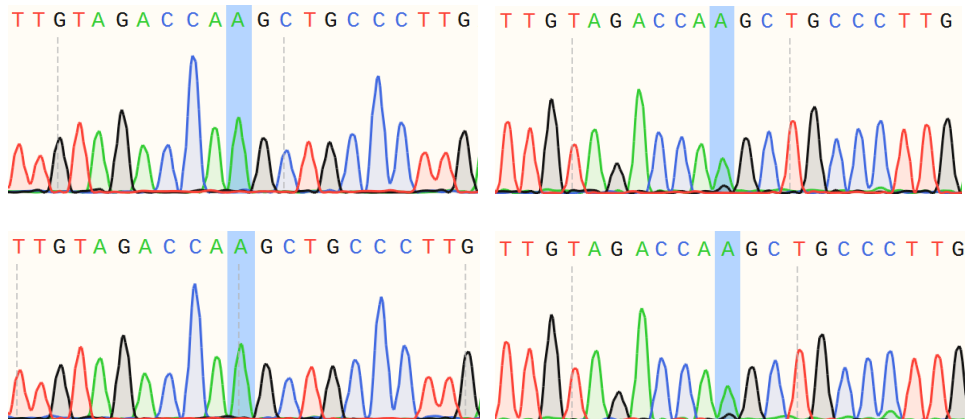

DNA

RNA

*Padi2:chr4:140951612*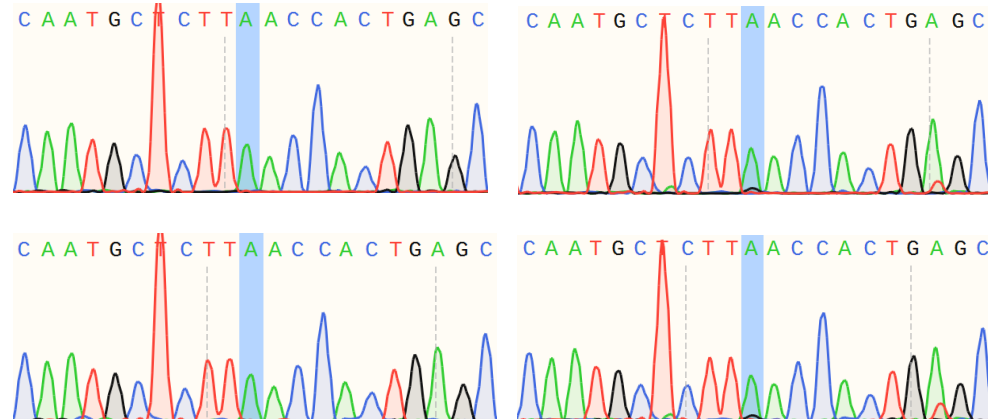*Rgs9bp:chr7:35581747*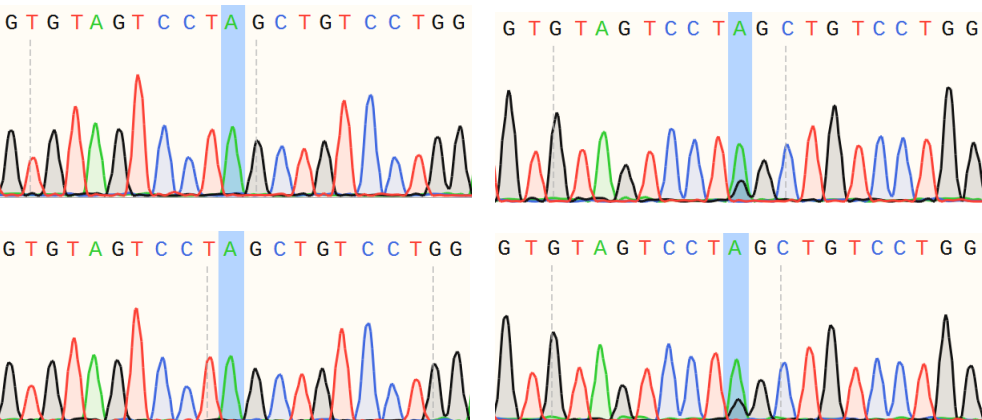*Calm1:chr12:100201186*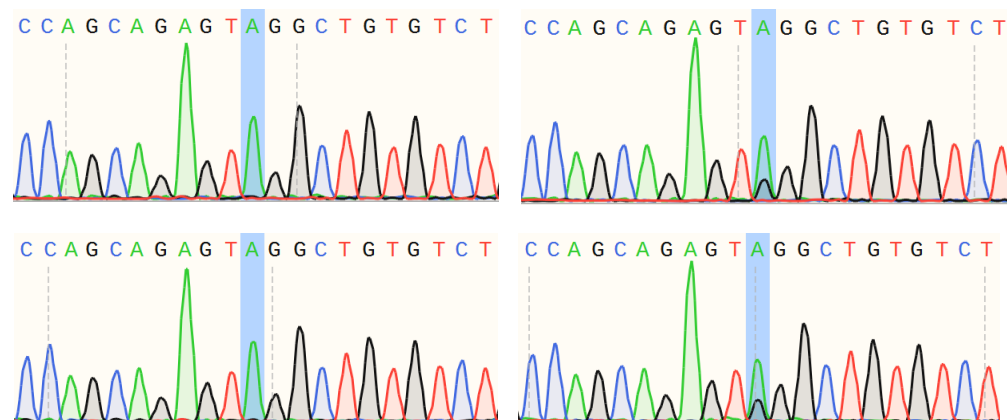

Supplement: Supplemental Information 2 — Four DESs (Rgs9bp: chr7:35581718, Rgs9bp: chr7:35581747, Padi2: chr4 :140951612 and Calm1: chr12:100201186) were validated with Sanger sequencing in two other different retina samples at DNA and RNA levels, respectively. [file peerj-14-21019-s002.pdf]
